# Grape Stem Extracts with Potential Anticancer and Antioxidant Properties

**DOI:** 10.3390/antiox10020243

**Published:** 2021-02-05

**Authors:** Javier Quero, Nerea Jiménez-Moreno, Irene Esparza, Jesús Osada, Elena Cerrada, Carmen Ancín-Azpilicueta, María Jesús Rodríguez-Yoldi

**Affiliations:** 1 Department of Pharmacology and Physiology, Veterinary Faculty, University of Zaragoza, 50013 Zaragoza, Spain; javierquero94@gmail.com; 2 Department of Science, Public University of Navarra, Institute for Advanced Materials and Mathematics (INAMAT^2^), 31006 Pamplona, Navarra, Spain; nerea.jimenez@unavarra.es (N.J.-M.); irene.esparza@unavarra.es (I.E.); 3 Department of Biochemistry and Molecular Cell Biology, Veterinary Faculty, University of Zaragoza, 50013 Zaragoza, Spain; josada@unizar.es; 4 CIBERobn, ISCIII, IIS Aragón, IA2, 50009 Zaragoza, Spain; 5 Department of Inorganic Chemistry, Sciences Faculty, University of Zaragoza, 50009 Zaragoza, Spain; ecerrada@unizar.es

**Keywords:** cancer cells, polyphenols, grape stem, proteasome, ROS, TrxR1

## Abstract

The application of plant extracts for therapeutic purposes has been used in traditional medicine because plants contain bioactive compounds with beneficial properties for health. Currently, the use of these compounds that are rich in polyphenols for the treatment and prevention of diseases such as cancer, diabetes, and cardiovascular diseases, many of them related to oxidative stress, is gaining certain relevance. Polyphenols have been shown to have antimutagenic, antioxidant, and anti-inflammatory properties. Therefore, the objective of the present work was to study the potential effect of grape stem extracts (GSE), rich in phenolic compounds, in the treatment of cancer, as well as their role in the prevention of this disease associated with its antioxidant power. For that purpose, three cancer lines (Caco-2, MCF-7, and MDA-MB-231) were used, and the results showed that grape stem extracts were capable of showing an antiproliferative effect in these cells through apoptosis cell death associated with a modification of the mitochondrial potential and reactive oxygen species (ROS) levels. Additionally, grape stem extracts showed an antioxidant effect on differentiated intestinal cells that could protect the intestine from diseases related to oxidative stress. Therefore, grape extracts contain bioactive principles with important biological properties and could be used as bio-functional food ingredients to prevent diseases or even to improve certain aspects of human health.

## 1. Introduction

Grape stems are by-products generated in great quantity in the winemaking process, and their elimination causes environmental problems. Therefore, it is important to find strategies that allow the reuse of these products. This residue is a rich source of phenolic compounds, celluloses, hemicelluloses, and lignins [1,2,3,4,5,6]. Among them, phenolic compounds confer antioxidant properties to the extracts obtained from grape stems. For this reason, different studies have been conducted in order to determine the polyphenolic composition of grape stems, and several proanthocyanidins, anthocyanidins, flavonols, hydroxycinnamic acids, and stilbenes have been found. Among them, the most characteristic polyphenolic substances referred to in most of the studies are *trans*-resveratrol, ε-viniferin, caftaric acid, gallic acid, catechin (one of the most abundant polyphenols), epicatechin, malvidin derivatives, quercetin, and glycosylated derivatives of quercetin in position 3 [2,3,5,7].

According to current research results, grape stem extracts possess important biological activities with multiple benefits for human health due to antioxidant and anti-inflammatory properties [5,7,8,9,10,11]. Veskoukis et al. [10] found that this by-product is particularly rich in flavonoids and stilbenes, such as *trans*-resveratrol and viniferin, which are found in considerably high concentrations. These authors also found that such extracts exhibited significant antioxidant properties, and, even at low concentrations, they showed a strong ability to prevent the oxidation of low-density lipoprotein (LDL) and to reduce intracellular levels of reactive oxygen species (ROS). In this way, Gonzalez-Centeno et al. [12] and Veskoukis et al. [10] evaluated the total phenolic and total proanthocyanidin composition of different grape stem varieties, as well as their antioxidant potentials. Grape stem extracts also prevent ROS-induced DNA damage and have inhibitory activity against liver and cervical cancer cell growth, suggesting their potential as chemopreventive agents [13]. Using human epidermal keratinocytes, Domínguez-Perles et al. [14] observed a protective effect of grape stem extracts against oxidative stress. These researchers found a close correlation between the concentration of phenolic compounds in the extracts and the potential to regulate the redox balance in vitro, as well as the capacity of these extracts to efficiently modulate apoptosis in HaCaT keratinocytes. Cho et al. [15] studied the effect of the topical administration of grape stem extracts to mice skin before subjecting them to UVB radiation for three minutes, thrice a week for one month. These authors demonstrated that these extracts significantly inhibited oxidative damage induced by UVB radiation and observed decreased epidermal hyperplasia, melanin pigmentation, and collagen degradation in the skin of mice. In addition, grape pomace—consisting of peel, seed, stem, and pulps—is discarded during grape processing, including juice extraction and winemaking, despite its substantial phenolic content [8]. In this way, Del Pino-Garcia et al. [16] studied the chemopreventive potential of powdered red wine seasonings against colorectal cancer in HT-29 cells. Grape seed extracts have also been applied as photochemopreventive agents against UVB-induced skin cancer [17]. Likewise, *Vitis vinifera* extracts have shown an antidiabetic effect by inhibiting the enzyme glycogen phosphorylase [18].

On the other hand, grape stem extracts have significant antimicrobial activities that seem to be influenced by the structure and function of phenolic compounds, as well as by their interspecific relation with different bacterial strains [19,20]. For example, these types of extracts inhibited the growth of both Gram-positive (*Listeria monocytogenes*, *Staphylococcus aureus* and *Enterococcus faecalis*) and Gram-negative *(Pseudomonas aeruginosa*, *Escherichia coli* and *Klebsiella pneumoniae*) digestive pathogens under in vitro conditions [21], and their bioactive compounds are used in oral care [22].

In addition, the extracts could be used to control the presence of human pathogenic bacteria in fresh leafy vegetables [6]. Leal et al. [23] studied the potential of grape stem extracts from different white grape varieties as antimicrobial agents to reduce the use of antibiotics. These authors found that the bactericidal activity of the extracts was higher, in general, against Gram-positive than Gram-negative bacteria, although they used a different methodology from that of Dias et al. [21] to evaluate the antimicrobial activity (minimum inhibitory concentration vs. disc diffusion). Other studies have shown that grape stem extracts are highly effective against foot wound ulcers produced by Gram-positive bacteria, and they also have anti-inflammatory action, inhibiting the production of nitric oxide lipopolysaccharide-stimulated macrophages by up to 35.25% [24].

During the last few years, our research group has investigated the chemopreventive properties of extracts obtained from different plant matrices such as rosehips, fenugreek, pine bark, and artichoke waste on human colon cancer [3,25,26,27]. Though there have only been few publications on this subject, the anticarcinogenic potential of grape stem extracts has also been studied in different cell lines [13,28,29]. Additionally, grape stem extracts possess important bioactivities such as antiangiogenic properties [30].

However, the phenolic composition—and therefore the biological activity and efficacy—of a specific grape stem extract depends on the procedure used to obtain the extract. In a previous study, we selected an optimized extraction method for grape stems [3], and the extracts obtained by this method presented high antioxidant potential and were demonstrated to be good candidates for SO_2_ substitution in wines [4]. With this background, the aim of the present research was to complete the characterization of those grape stem extracts by studying their potential for the treatment of human colorectal adenocarcinoma (Caco-2) and human breast adenocarcinoma (MCF-7 and MDA-MB-231) cell lines. Thus, we measured the possible antiproliferative effects of these extracts on cancer cells and their mechanisms of action. Furthermore, the protective effects of these extracts, in a model of intestinal barrier (differentiated Caco-2 cells), were also tested through the measurement of the intracellular levels of ROS.

## 2. Materials and Methods

### 2.1. Extracts

The grape stem extract was obtained through an extraction method using GRAS solvents from Mazuelo-variety stems harvested in the 2016 vintage [3]. Briefly, grape stems were oven-dried at 25 °C, ground, and sieved (ϕ < 0.3 mm). The extract was obtained after macerating the ground and sieved stems in 50% ethanol/water, with a 1:100 (*w/v*) ratio and at 40 °C for 24 h. Then, the extract was centrifuged (8000 rpm for 15 min), filtered through filter paper, and lyophilized (Telstar Cryodos freeze drier, Madrid, Spain).

### 2.2. Chemicals

All the used HPLC solvents were from Scharlab (Barcelona, Spain). All the used phenolic standards were from Sigma-Aldrich (Madrid, Spain), with the exception of malvidin-3-glucoside (enyn-chloride, Extrasynthese, Genay, France). Among the chemicals for spectrophotometric analysis, the Folin–Ciocalteu reagent, Trolox (6-hydroxy-2,5,7,8-tetramethylchroman-2-carboxylic acid), gallic acid, and quercetin were supplied by Sigma-Aldrich (Madrid, Spain); glacial acetic acid, anhydrous sodium carbonate, and aluminum chloride 6-hydrate were supplied by PanReac AppliChem (Barcelona, Spain).

### 2.3. Identification and Quantification of Phenolic Composition of Grape Stem Extracts by HPLC-DAD

The identification and quantification of the phenolic compounds present in the grape stem extracts were performed using high-performance liquid chromatography. The chromatograph was equipped with two 510 pumps, a 717 Plus autosampler, and a 996 photodiode array detector (Waters Div., Milford, MA, USA). A Zorbax Eclipse Plus C18 reversed phase column (250 × 4.6 mm; particle size of 5 µm) (Agilent, Santa Clara, CA, USA) was used. For the analyses of the extract, between 45.0 ± 0.1 and 70.0 ± 0.1 mg of each sample were weighted and dissolved in 10 mL of methanol with the aid of an ultrasonic bath (JP Selecta, Barcelona, Spain). Samples were prepared in triplicate and analyzed once. The chromatographic analyses were carried out according to a modified method of Barros et al. [31]. Two mobile phases, A (water: 85% formic acid, 99:1 *v/v*) and B (acetonitrile: 85% formic acid, 99:1 *v/v*) were used. The flow rate was 1 mL/min using the following linear gradient scheme (t in min; % A): 0, 95%; 15, 85%; 22, 80%; 25, 80%; 35, 70%; 45, 50%; 50, 5%; 55, 95%; and 60, 95%. The column temperature was 30 °C, and the injection volume was 40 μL. The identification of the different compounds was performed by the double coincidence of the retention time of its corresponding standard and the UV–Vis spectrum of each compound. Quantification was carried out using calibration curves for each analyzed compound. The calibration curves used for resveratrol, gallic acid, quercetin, malvidin-3-glucoside, and caftaric acid presented linear correlation coefficients higher than 0.999. The calibration curves obtained for the rest of compounds (viniferin, catechin, and the derivative of quercetin) showed linear correlation coefficients higher than 0.998. In the case of the unidentified anthocyanin, it was not possible to identify its structure with the method used in the laboratory. However, most of the anthocyanins described in the literature that are present in grape stems correspond to derivatives of malvidin. For this reason, and given the fact that all anthocyanins have similar general structures, we used the calibration curve of malvidin-3-glucoside to estimate the concentration of the unknown anthocyanin.

### 2.4. Determination of Antioxidant Capacity of the Grape Stem Extracts by DPPH

The DPPH (2,2-diphenyl-1-pycrilhydracyl) assay was based on the method proposed by Brand-Williams et al. [32]. A standard solution of 24 mg of DPPH in 100 mL of methanol was prepared, and then it was diluted in methanol until we obtained an absorbance of 0.9±0.1 at 517 nm in a UV–Vis spectrophotometer (Jenway, Staffordshire, UK). For the calibration curve, seven different Trolox standards were prepared in methanol in concentrations from 0.05 to 0.73 mM. For sample preparation, between 50.0 ± 0.1 and 72.0 ± 0.1 mg of extract were dissolved in 10 mL of methanol, and the resulting mixture was diluted 10 times with methanol. For analysis, 150 µL of the Trolox standard solution or processed sample were mixed with 2.85 mL of the DPPH solution. After 30 min in darkness, the antioxidant capacities of all the standards and samples were determined by measuring the absorbance at 517 nm. For each batch of extract, three different processed samples were prepared, and each of them was analyzed once. The linear correlation coefficient obtained for the calibration curve was R^2^ > 0.998. The results of antioxidant capacity were expressed as mmol Trolox/g of extract.

### 2.5. Spectrophotometric Determination of Total Phenolic and Flavonoid Content of the Grape Stem Extracts

Total phenolic content was analyzed using the Folin–Ciocalteu method, as described by Singleton et al. [33]. For the calibration curve, different gallic acid standards were prepared in methanol in concentrations from 0.2 to 4.6 mM. For sample preparation, between 50.0 ± 0.1 and 72.0 ± 0.1 mg of extract were dissolved in 10 mL of methanol. For analysis, 100 µL of the gallic acid standard solution or processed sample were mixed with 0.5 L of the Folin–Ciocalteu reagent, 7.9 mL of deionized water, and 1.5 mL of Na_2_CO_3_ (20% *w/w*), and the resulting solutions were left for 2 h in darkness. The absorbance was measured at 765 nm in a UV–Vis spectrophotometer (Jenway, Staffordshire, UK). The standard used for the calibration curve was gallic acid, ranging between 0.2 and 5.08 mM. The linear correlation coefficient obtained for the calibration curve was R^2^ > 0.999. For each batch of extract, three different processed samples were prepared, and each of them was analyzed once. The results of total phenolic content were expressed as mg gallic acid/g extracts.

The total flavonoid content was determined by the colorimetric method of aluminum chloride using a solution of 2% AlCl_3_ in 5% acetic acid [34]. For the calibration curve, different quercetin standard solutions were prepared in methanol in concentrations from 3 to 30 µg/mL. For sample preparation, between 50.0 ± 0.1 and 72.0 ± 0.1 mg of extract were dissolved in 10 mL of methanol. For analysis, 1.5 mL of the quercetin standard solution or sample were mixed with 1.5 mL of the AlCl_3_ solution, and the resulting solutions were left for 30 min in darkness. Then, absorbance was measured on a Jenway UV–Vis spectrophotometer at 420 nm. The linear correlation coefficient obtained for the calibration curve was R^2^ > 0.999. For each batch of extract, three different processed samples were prepared, and each of them was analyzed once. The results were expressed as mg of quercetin/g extracts. In all cases, the samples were analyzed in triplicate.

### 2.6. Cell Culture

Human Caco-2 cell line (TC7 clone) was kindly provided by Dr. Edith Brot-Laroche (Université Pierre et Marie Curie-Paris 6, UMR S 872, Les Cordeliers, France). Human breast adenocarcinoma MDA-MB-231 cells were kindly provided by Dr. Carlos J. Ciudad and Dr. Verònica Noé (Departamento de Bioquímica y Fisiología, Facultad de Farmacia, Universidad de Barcelona, Spain). Human breast adenocarcinoma MCF-7 cells were kindly provided by Cristina Sanchez-de-Diego (Departamento de Fisiología II, Universidad de Barcelona, Spain). Human fibroblast cells were kindly provided by Dr. Julio Montoya (Departamento de Bioquimica, Universidad de Zaragoza, Spain). All cell lines were maintained in a humidified atmosphere of 5% CO_2_ at 37 °C. Cells were grown in Dulbecco’s Modified Eagle Medium (DMEM) supplemented with 20% fetal bovine serum (FBS), 1% non-essential amino acids, 1% penicillin (1000 U/mL), 1% streptomycin (1000 μg/mL), and 1% amphotericin (250 U/mL). The cells were enzymatically passaged with 0.25% trypsin–1 mM EDTA and sub-cultured on 25 cm^2^ plastic flasks at a density of 5 × 10^5^ cells/cm^2^. The culture medium was replaced every 2 days. Extract treatments were added 24 h post-seeding for assays on undifferentiated Caco-2 cells [35] and 10–15-days post-seeding on differentiated Caco-2, MCF-7, and MDA cells. Cell confluence (80%) was confirmed by optical microscopy observance.

### 2.7. Cell Treatment and Antiproliferative Property Analysis

Extracts from grape stems were diluted in a cell culture medium to a final concentration 1.5 mg/mL. For cytotoxicity screening assays, the cells were seeded in 96-well plates at a density of 4 × 10^3^ cells/well. The culture medium was replaced with a medium containing plant extracts, and cells were incubated for 48 or 72 h. The antiproliferative effect was measured with the sulforhodamine B (SRB) assay, as previously described [36]. Absorbance at 540/620 nm was measured with the SPECTROstar Nano (BMG Labtech, Ortenberg, Germany). The effect on cell growth was expressed as a percentage of the control. Finally, the IC_50_ value was calculated under all conditions tested. IC_50_ represents the concentration of compound that halves cell proliferation or viability. This value was selected for further analysis to elucidate the extracts’ mechanism of action on cancer cells.

### 2.8. Measurements of Apoptosis

The cells were seeded in 25 cm^2^ flasks (5 × 10^5^ cells/cm^2^), exposed to plant extracts for 48 h at the IC_50_ concentration, and then collected and stained with annexin V-FITC and propidium iodide, as previously described [37]. A negative control was prepared by untreated cells, and it was used to define the basal level of apoptotic and necrotic or dead cells. After incubation, cells were transferred to flow cytometry tubes and washed twice with phosphate-buffered saline (PBS), followed by a resuspension in 100 µL of the annexing V binding buffer (100 mM HEPES/NaOH pH 7.4, 140 mM NaCl, and 2.5 mM CaCl_2_). To each tube, 5 µL of annexin V-FITC and 5 µL of propidium iodide were added. After 15 min of incubation at room temperature in the dark, 400 µL of the annexin binding buffer were added and analyzed by flow cytometry within 1 h. The signal intensity was measured using a BD FACSAria^TM^ cell sorter (BD Biosciences, San Jose, CA, USA) and analyzed using the BD FASCDiva^TM^ software (BD Biosciences, San Jose, CA, USA).

### 2.9. Flow Cytometry Mitochondrial Membrane Potential Assay

Cells were seeded in 25 cm^2^ flasks and then exposed to plant extracts for 48 h. The control cells were incubated with a new medium without treatment. Then, cells were washed twice with PBS. The pellet was resuspended in PBS at concentration of 10^6^ cell/mL, and 5 μL of 10 μM 1,1′,3,3,3′-hexamethylindodicarbo-cyanine iodide (DiIC1) were added to each sample. Tubes were incubated at 37 °C for 15 min, and 400 μL of PBS were added prior to analyze fluorescence with BD FACSarray^TM^ (BD Biosciences, San Jose, CA, USA) equipped with an argon ion laser. The excitation and emission settings were 633 and 658 nm, respectively [37].

### 2.10. Determination of Intracellular Levels of Reactive Oxygen Species (ROS)

The cells were seeded in 96-wells plate at a density of 4 × 10^3^ cells/well. The intracellular level of ROS was assessed using the dichlorofluorescein assay, as previously described [37]. Cells were cultured 24 h before being incubated with stem extracts and then underwent oxidative stress induction by adding H_2_O_2_ (80 mM) for 20 min. After that, the medium was removed, cells were washed twice with PBS, and cells were incubated for 1 h with 20 μM 2′,7′-dichlorofluorescein diacetate (DCFH-DA) in PBS at 37 °C. The formation of the fluorescence oxidized derivative of DCF was monitored at an emission wavelength of 535 nm and an excitation of 485 nm in a FLUOstar Omega (BMG Labtech, Ortenberg, Germany) multiplate reader. A measure at time “zero” was performed, cells were incubated at 37 °C in the multiplate reader, and the generation of fluorescence was measured after 20 min. ROS levels were expressed as a percentage of fluorescence compared to the control. The obtained values of fluorescence intensity are considered as a reflection of total intracellular ROS content.

### 2.11. Determination of Proteasome Activity

The cells (5 × 10^5^ cells/cm^2^) were seeded in a cell culture flask (25 cm^2^). The determination of the proteasome activity was carried out with a fluorometric assay using a proteasome 20S activity assay kit (MAK172, Sigma-Aldrich, Madrid, Spain) based on Suc-LLVy-AMC, a fluorogenic substrate of the proteasome β5 submit. Caco-2 cells were treated with grape stem extracts for 24 h post-seeding and then processed following instructions in the kit protocol. The fluorescence levels correspond to the proteasomal chymotrypsin-like activity (CT-L activity). The activity was measured in lysed cells with FLUOstar Omega (BMG Labtech, Ortenberg, Germany), and the value was obtained per mg of protein. The data are expressed in % CT-L activity.

### 2.12. Thioredoxin Reductase 1 (TrxR1) Activity

The cells were seeded in a 96-well plate with grape stem extracts for 24 h. The cells were then lysed and incubated with a shaking motion for 20 min before adding 25 µL/well of the reaction buffer (500 μL of PBS pH 7.4, 80 μL of 100 mM EDTA pH 7.5, 20 μL of 0.05% BSA, 100 μL of 20 mM NADPH, and 300 μL of distilled H_2_O), and the reaction was started with DTNB (20 mM in pure ethanol), as previously described by Allaoui et al. [26]. The absorbance increase was followed at 405 nm with SPECTROstar Nano (BMG Labtech, Ortenberg, Germany) every minute for 6 min. The value was obtained per mg of protein and expressed in % thioredoxin reductase (TrxR) activity.

### 2.13. Statistical Analysis

All assays were performed at least three times. Data are presented as mean ± SD. Means were compared using ANOVA. Significant differences at *p* < 0.05 were compared using a Bonferroni’s multiple comparison test. The statistical analyses were performed and the graphics were obtained using the GraphPad Prism Version 5.02 software for Windows (GraphPad Software San Diego, CA, USA).

## 3. Results and Discussion

Plant polyphenols represent a variety of bioactive compounds that are capable of preventing and controlling cancer and diabetes, as well as neurodegenerative, autoimmune, cardiovascular, and ophthalmic diseases [38]. The presence of polyphenol compounds in grape stems gives them exceptional biological value [39]. It has been shown that extracts derived from grape stems possess potent antioxidant activity in vitro [40] and in cell lines [7], whereas their anticarcinogenic role has not been widely reported.

The present study investigated the biological properties of grape stem extracts on different cancer cells, as well as their mechanisms of action. Furthermore, the extracts’ effects on the prevention of oxidative stress in a model of differentiated intestinal cells was also studied.

### 3.1. Phenolic Composition and Antioxidant Activity in Mazuelo Stem Extracts

The phenolic composition, as well as the total polyphenol and flavonoid contents, of Mazuelo stem extracts are presented in Table 1. In this extract, nine phenolic compounds were found, of which the most abundant were (+)-catechin and the quercetin-3-derivative. Likewise, Leal et al. [24] found that (+)-catechin was the most abundant phenolic compound in Portuguese grape stem extracts from different varieties (Tinta Roriz, Touriga Nacional, Castelão, Syrah, Arinto, and Fernão Pires). Anastasiadi et al. [11] also reported the presence of several phenolic compounds in grape stem extracts from six red and white varieties from Greece. In comparison to their results with our Mazuelo stem extract, *trans*-resveratrol, ε-viniferin, (+)-catechin, and caftaric acid coincide, (+)-catechin was found to be the most abundant in both studies. Regarding the concentrations of resveratrol and viniferin, these authors observed differences among varieties and vintages. Lambert et al. [41] analyzed the stilbene content of pruning canes of the Carignan variety, which is the name given in France to the Mazuelo variety. These authors found a higher amount of resveratrol and viniferin in their extracts (0.88 mg resveratrol/g extract and 0.97 mg viniferin/g extract), although it must be considered that grapevine canes are probably richer in stilbenes than grape stems [42,43]. In addition to the phenolic compounds found in the Mazuelo stems analyzed in this work, other compounds have been identified in grape stem extracts of different varieties. For instance, in stem extracts from Portuguese grapes, kaempferol and isorhamnetin were identified [2], and in grape stems from Greek varieties, ferulic, coumaric, caffeic, and syringic acids were identified [28].

Regarding the antioxidant capacity measured by the DPPH assay (Table 1), the result of the Mazuelo stem extract was similar to that of the Syrah (0.44 ± 0.04 mmol Trolox/g) and Fernão Pires (0.55 ± 0.01 mmol Trolox/g) extracts and higher than that of the Castelão (0.31 ± 0.01 mmol Trolox/g) and Arinto (0.15 ± 0.01 mmol Trolox/g) varieties found by Leal et al. (2020).

### 3.2. Effect of Extracts From Grape Stem on Cancer Cells

#### 3.2.1. Antiproliferative Activity

**Figure 1:**
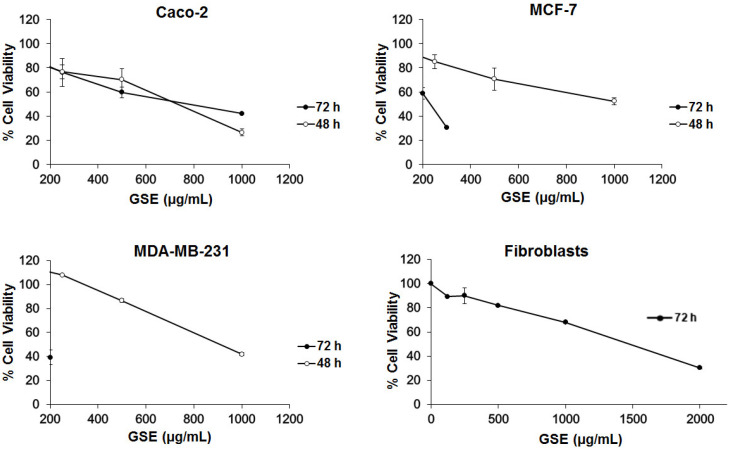
Measurement of Caco-2, MCF-7, MDA-MB-231, and fibroblast cell viability at 48 and 72 h after incubation with grape stem extracts (GSE). The GSE concentrations tested in the four types of cells were 62.5, 125, 250, 500, and 1000 μg/mL, but at 72 h in the MCF-7 and MDA-MB-232 cells, the chosen concentrations were 9, 18, 37.5, 75, and 200 μg/mL.

The toxicity of extracts from grape stems was evaluated on undifferentiated Caco-2, MCF-7, and MDA-MB-231 cells by an SRB assay. Initially, a range of concentrations of grape extracts (62.5, 125, 250, 500, and 1000 μg/mL) was tested. The concentrations chosen were in relation to previous work carried out by our research group with other plant extracts [25,36]. The IC_50_ was calculated in the different cell lines at two time-points of 48 and 72 h. However, in the MDA-MB-231 and MCF-7 cells, when treated for 72 h, this range was lethal in most concentrations and the IC_50_ could not be calculated, so the range was modified to decreased concentrations (range: 9, 18. 37.5, 75, and 200 μg/mL). These results suggest that cytotoxic effect of grape stem extracts (GSE) is concentration- and time-dependent and that Caco-2 cells are less sensitive to GSE at the highest incubation time. At 48 h, similar viability curves were obtained in the three different cell lines (Figure 1, Table 2).

The results showed that the grape stem extracts were not selective for a single cancer line, but they produced a decrease in viability in the three tested cell lines (Figure 1). The effect was faster in Caco-2 cells, although their effectiveness was greater in breast cells (MCF-7 and MDA-MB-231) at longer times (72 h). In order to determine the action of these extracts on a noncancerous model, the IC_50_ was calculated on human fibroblast cells, after 72 h of incubation, where we observed a significantly lower effect. These data could be used to obtain a selectivity index (SI), as previously described by Badisa el al. [44]. The SI results are shown in Table 2, with the highest value being for the MDA-MB-231 cell line, according with the highest effective response of the extracts towards these cells after 72 h of incubation. The observed difference in the two breast cancer lines could have been due to the fact that the action of these extracts could be related to the receptors’ expression for estrogens, which are only present in MCF-7 cells [45].

#### 3.2.2. Cell Death Studies

**Figure 2:**
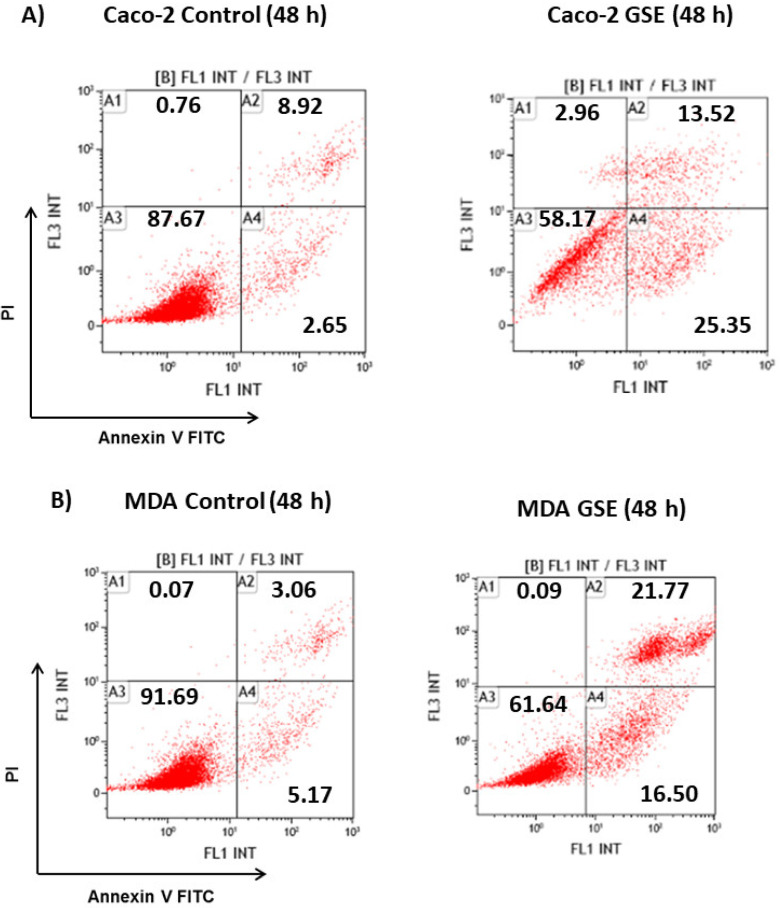

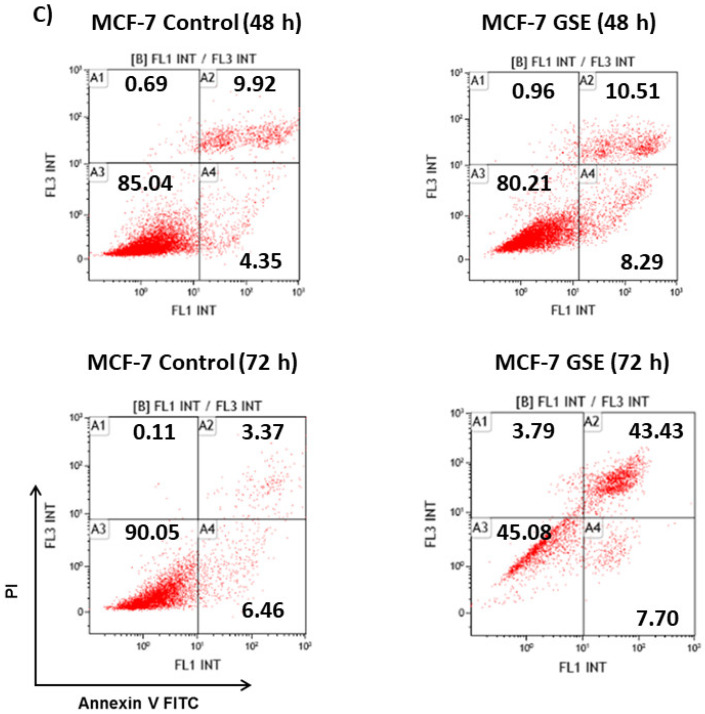
Analysis of the type of cell death induced on Caco-2 (**A**) MCF-7 (**B**), and MDA-MB-231 (**C**) after 48 or 72 h of incubation in control (untreated cells) and grape stem extracts (GSE) at IC_50_ (μg/mL) on Caco-2 (759), MCF-7 (203), and MDA-MB-231 (85). Percentages of live (A3), necrotic (A1), early apoptotic (A4), and late apoptotic (A2) cells are indicated.

Since the grape stem extracts produce a reduction in cell viability, it was decided to determine what type of cell death occurred. Thus, flow cytometry analyses over 48 h were performed using biomarkers of cell death. The results showed that treatment for 48 h with the IC_50_ concentration corresponding to each cell line mainly produced early apoptosis in Caco-2 cells, while late apoptosis was mainly detected in MDA cells. However, no significant apoptosis was found in MCF-7 cells. Treatment with longer time (72 h) induced a significant death of these cells by late apoptosis (Figure 2). Therefore, the obtained results showed that grape stem extracts at their IC_50_ produced apoptosis in all tested cancerous cells by activating apoptotic pathways, thereby reducing their ability to non-selectively react with biological targets to cause necrosis and its related side effects.

**Figure 3:**
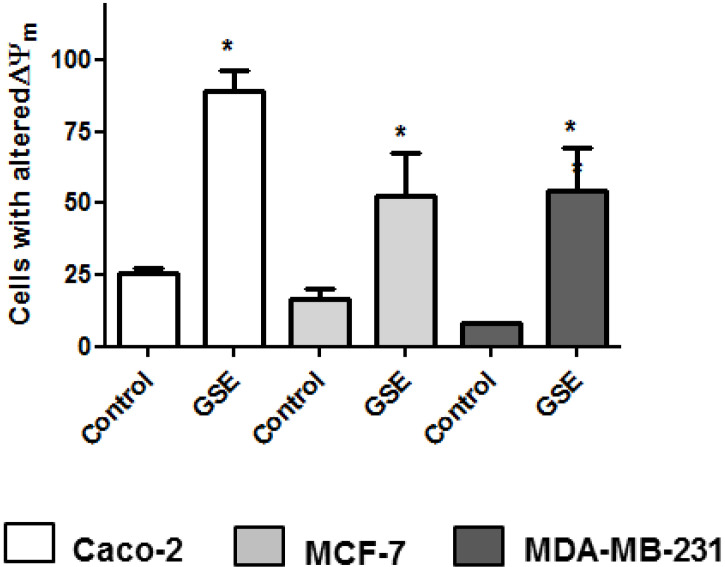
Analyses of mitochondrial membrane potential (∆Ψm) after 48 h of incubation with grape stem extracts (GSE) at their IC_50_ (μg/mL) in Caco-2 (759), MCF-7 (203), and MDA-MB-231 (85). * *p* < 0.05 vs. respective control (untreated cells).

Since previous studies on plant extracts suggested mitochondrial dysfunction and intrinsic apoptosis induction [25,26], the mitochondrial membrane potential change was analyzed. Mitochondria play a pivotal role in life and cell death inasmuch as they produce the majority of the energy required for survival and regulate the intrinsic apoptosis pathway. The involvement of mitochondria in cell death is generally measured by following mitochondrial membrane depolarization [46]. The results showed that grape stem extracts significantly altered the mitochondrial potential of the tested cancer cells compared to the untreated ones (Figure 3); therefore, the changes in mitochondrial potential could be related to the observed apoptosis (Figure 2).

#### 3.2.3. ROS Intracellular Levels

**Figure 4:**
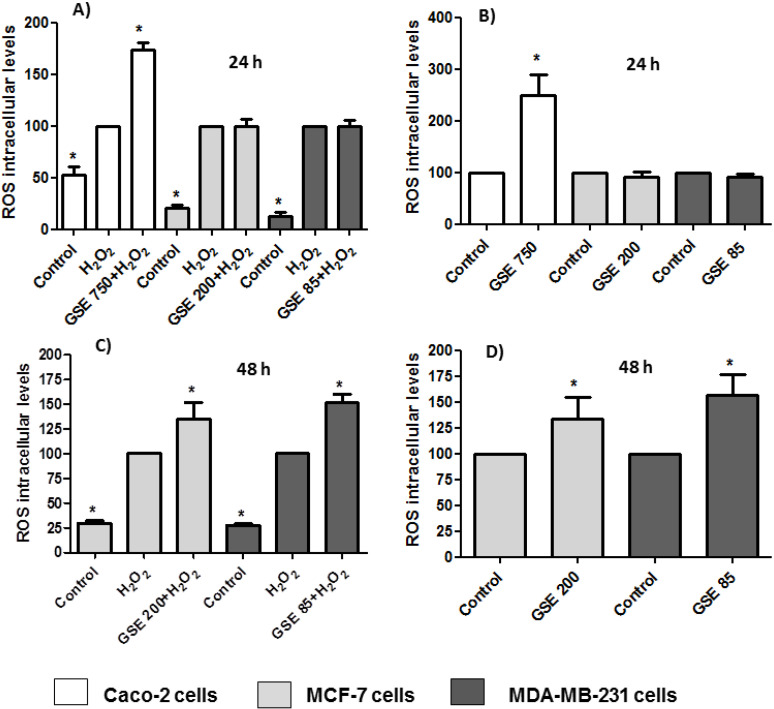
Measurements of reactive oxygen species (ROS) levels in the presence (**A**,**C**) or absence of H_2_O_2_ (80 mM, 20 min) (**B**,**D**) after 24 or 48 h of incubation with grape stem extracts (GSE) at IC_50_ (μg/mL) in Caco-2 (759), MCF-7 (203), and MDA-MB-231 (85). * *p* < 0.05 vs. respective control (untreated cells) (**B**,**D**) or vs. H_2_O_2_ (**A**,**C**).

The oxidative stress imposed by ROS plays an important role in many chronic degenerative diseases and cancers. Higher levels of ROS are generated through an increase in metabolic activity of cancer cells including enhanced signaling pathways or mitochondrial dysfunction [47]. The ROS levels in the cells were determined based on the reaction between ROS and DCFH-DA. The assays were carried out by treating the cells with the grape stem extracts in the presence or absence of H_2_O_2_. Hydrogen peroxide is a widespread substance used to mimic the pro-oxidative environment that characterizes degenerative diseases such as cancer or neurodegenerative disorders on 2D cell cultures. The results showed that in Caco-2 cells, the extracts at a concentration of 750 μg/mL (IC_50_) were able to show a pro-oxidant effect after 24 h in both the absence and presence of hydrogen peroxide (Figure 4A,B). Therefore, this increase in oxidative stress caused by grape stem extracts, together with the significant change in the potential of the mitochondrial membrane, seems to be the cause of Caco-2 cell death by apoptosis. However, it must be considered that at low extract concentrations, a slight tendency to produce an antioxidant effect was observed, but there was no significant antiproliferative effect (data not shown). No modification of ROS levels was found in breast cells when they were treated with extracts at their respective IC_50_ and lower concentrations after 24 h (Figure 4A,B). Since breast cells seem to show a slower response to treatment with extracts after 24 h, (Table 2 and Figure 2) and previous results had shown a change in mitochondrial potential at 48 h (Figure 3), the ROS levels were measured at this time. The results showed a significant increase of ROS levels in both conditions (with/without H_2_O_2_) (Figure 4C,D).

The antioxidant effect of polyphenols has been extensively studied [38], although they may also have a pro-oxidant effect. These results have been mainly observed in tumor cells and have been related to pro-apoptotic action. The dual pro-oxidant and antioxidant behavior of phenolic plant compounds depends not only on the cell type but also on their concentration, chemical structure, and pH status [48,49].

#### 3.2.4. Proteasome Activity

The ubiquitin/proteasome system (UPS) is a complex molecular machinery that constitute the main proteolytic pathway in eukaryotic cells. The UPS is involved in the regulation of basic biological process such as cell growth, proliferation, cell cycle, and apoptosis [50], and the dysregulation of these processes causes malignant transformation. Therefore, several cancer cells have a dysfunctional UPS with an increased activity of the proteasome [51], and various studies have shown that the inhibition of the proteasome in cancer cells may lead to the accumulation of inhibitors of cyclin-dependent kinases, pro-apoptotic proteins, and tumor suppressor proteins, leading to programmed cell death or apoptosis [52,53].

NF-kB proteins in the cytoplasm are associated with inhibitory proteins known as IkBs. The main activated form of NK-kB is a heterodimer composed of p65 and p50 subunits. NF-kB activation involves the phosphorylation of IkBs, after which it is ubiquitinated and degraded by the proteasome. Then the resulting free NF-kB is translocated to the nucleus, where it binds to kB-binding sites in the DNA and induces the transcriptions of several mediators.

**Figure 5:**
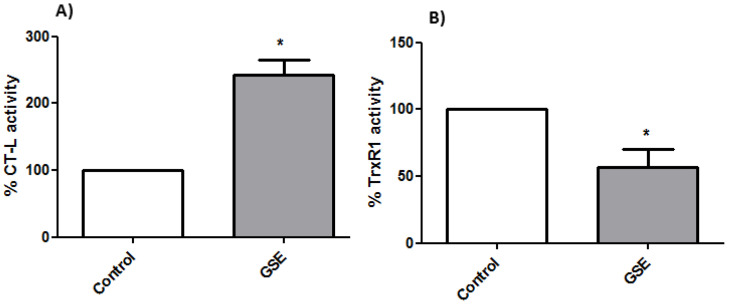
(**A**) Determinations of proteasomal chymotrypsin-like (CT-L) activity and (**B**) thioredoxin reductase 1 (TrxR1) activity from Caco-2 cells after 24 h of incubation with GSE to the IC_50_ concentration (759 μg/mL) * *p* < 0.05 vs. negative control (without treatment).

To analyze whether the grape stem extracts were able to interact with the proteasome, which is involved in the activation of the NF-kB factor and its translocation to the nucleus, its activity was determined by a fluorometric assay. The results showed an increase in the cells’ proteasomal CT-L activity after 24 h of treatment with grape stem extracts (Figure 5). ROS often stimulates the NF-kB pathway in the cytoplasm but inhibits NF-kB in the nucleus [54]. In the cytoplasm, ROS have been shown to activate NF-kB through the alternative phosphorylation of IkBα, which may or may not result in the degradation of IkBα. Furthermore, ROS can influence the DNA-binding properties of the NF-kB proteins themselves. The oxidation of p50 in its DNA-binding domain has been shown to prevent its binding to DNA and, therefore, the activation of the NF-kB factor [55]. Therefore, the increase in ROS levels produced by grape stem extracts could oxidize the p50 subunit of the NF-kB factor, thus inhibiting its binding to nuclear DNA and causing an upregulation of the proteasome (Figure 5A).

#### 3.2.5. TrxR1 Activity

The thioredoxin system is one of the most important antioxidant systems in mammalian cells, and it is constituted by thioredoxin (Trx), the enzyme TrxR, and NADPH. Though the principal function of the thioredoxin system is controlling intracellular redox homeostasis and repairing oxidative damage, it is also implicated in cell growth and apoptosis control [56]. The overexpression of Trx has been shown to diminish NF-kB activation by inhibiting IkB degradation and can reverse the inhibition of p50 DNA binding caused by an increased amount of ROS [57]. However, in the present study, TrxR1 activity was found to be lower in treated cells, and this fact could explain the high levels of ROS found after treating the cells with the extracts (Figure 5B).

Therefore, the effect of the extracts on the viability of Caco-2 cells seemed to be related to an increase in ROS levels by an inhibition of TrxR1 that indirectly caused an upregulation in the proteasome due to the inhibition of NF-kB activity.

### 3.3. Antioxidant Capacity of Grape Stem Extracts on a Model Intestinal Barrier

Considering the high antioxidant capacity and content of polyphenols found in the grape stem extracts measured by the DPPH assay (Table 1), and given that this effect at the IC_50_ concentration was not found in cancer cells, it seemed interesting to evaluate whether these extracts showed antioxidant capacity on a model of the intestinal barrier (differentiated Caco-2 cells) upon exogenous oxidative stress by hydrogen peroxide insult or in absence of H_2_O_2_. High intracellular ROS levels are related to the initiation, development, and progression of cancer, since free radicals lead to malignant transformation and damage lipids, proteins, and nucleic acids [58]. Therefore, testing the capacity of grape stem extracts to protect normal cells from oxidative stress by reducing ROS levels was an interesting way to elucidate whether GSE would be useful not only in cancer treatment but also in the prevention of cancer onset. Caco-2 cells spontaneously acquire the phenotypic features of non-cancerous enterocytes after reaching confluence (differentiated cells). Monolayer Caco-2 cells form tight junctions and present the cylindrical polarized morphology of enterocytes, expressing functional microvilli on the apical membrane [59,60,61]. Therefore, differentiated Caco-2 cells have been established as an acceptable in vitro intestinal barrier model.

**Figure 6:**
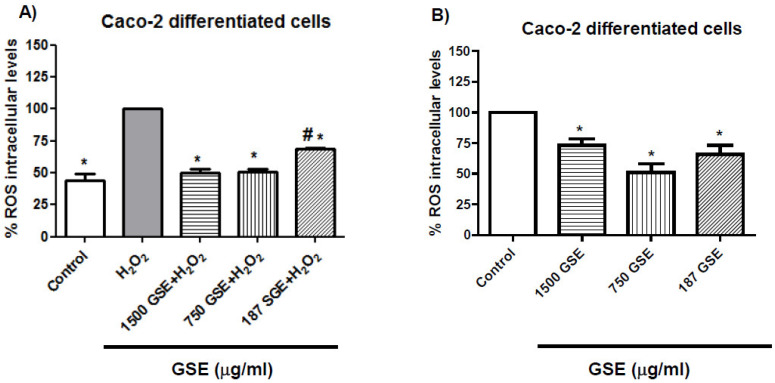
Measurements of ROS levels in the presence (**A**) or absence of H_2_O_2_ (50 mM, 1 h) (**B**) after 24 h of incubation with grape stem extracts (GSE) at 1500, 750, and 187 μg/mL. * *p* < 0.05 vs. respective H_2_O_2_, # *p* < 0.05 vs. respective control (untreated cells) (**A**), * *p* < 0.05 vs. respective control (untreated cells) (**B**).

In these differentiated cells, the antioxidant capacity of the extracts was managed at concentrations of IC_50_ (1500 μg/mL), previously obtained in fibroblasts (non-cancerous cells) and 750 and 187 μg/mL (1/2 and 1/8 IC_50_, respectively) in cancer cells, and they were tested for 24 h of incubation time. The results showed a clear antioxidant effect by decreasing the ROS level with/without H_2_O_2_-induced ROS production (Figure 6). In similar ways, plant extracts have been investigated in other studies for their capacity to correct the aberrant increase in ROS levels derived from H_2_O_2_ exogenous addition [62,63].

The antioxidant capacity of plant extracts is strongly correlated with their clinical application in gastrointestinal diseases related to oxidative stress [64]. These results obtained with grape extracts suggest that they could have potential applications in the management of gastrointestinal diseases related to oxidative stress.

## 4. Conclusions

In this work, the effects of grape stem extracts on cancer cells (Caco-2, MCF-7, and MDA-MB-231) and the intestinal barrier (differentiated Caco-2 cells) were studied. The extracts caused a decrease in the growth of cancer cells, causing death by apoptosis through the modification of mitochondrial potential and a decrease in the antioxidant enzyme TrxR1 that produces an increase in the cellular levels of ROS capable of inhibiting the binding of NF-kB to the nucleus and causing an upregulation of the proteasome. In the intestinal barrier, these extracts would produce an antioxidant effect, consistent with the antioxidant capacity found in the analysis of the extracts by DPPH, that would protect the intestine from disorders related to oxidative stress. For all these reasons, grape stem extracts might have a promising future in cancer treatment and the management of oxidative stress in the gastrointestinal tract. In addition, further research should be performed to analyze grape stem extracts’ potential as antioxidants and preservatives in food, as well as their beneficial role for health. This would help solve a waste accumulation problem, since the by-products of the agro-food industry could return to the circular economy, being used in food and pharmaceutical industries.

## Figures and Tables

**Table 1:** Phenolic composition (mg/g extract) and antioxidant capacity of the Mazuelo stem extract.

Phenolic Composition & Antioxidant Capacity	Grape Stem Extract
Gallic acid	0.21 ± 0.03
Caftaric acid	0.14 ± 0.03
(+)-Catechin	0.98 ± 0.20
Quercetin	0.05 ± 0.01
Quercetin-derivative ^1^	0.91 ± 0.08
Malvidin-3-glucoside	0.10 ± 0.02
Unknown anthocyanin ^2^	0.15 ± 0.02
*Trans*-resveratrol	0.26 ± 0.04
*Trans*-*ε*-viniferin	0.59 ± 0.09
Total phenolic content ^3^	83 ± 2
Total flavonoid content ^4^	2.6 ± 0.1
Antioxidant capacity by DPPH ^5^	0.47 ± 0.04

^1^ Expressed as quercetin-3-glucoside; ^2^ expressed as malvidin-3-glucoside; ^3^ expressed as mg gallic acid/g extract; ^4^ expressed as mg quercetin/g extract; ^5^ expressed as mmol Trolox/g extract.

**Table 2:** IC_50_ (the concentration of compound that halves cell proliferation or viability) values of grape stem extracts on Caco-2, MCF-7, MDA-MB-231, and fibroblast cells after 72 and 48 h of incubation.

	IC_50_ (µg/mL) 72 h	IC_50_ (µg/mL) 48 h	Selectivity Index
Caco-2	759 ± 51	661 ± 48	2.9
MCF-7	203 ± 53	817 ± 52 *	7.2
MDA-MB-231	85 ± 9	911 ± 10 *	17.0
Fibroblast	1454 ± 6	-	-

* *p* < 0.05; incubation time 48 vs. 72 h.
